# Supplementary material for: Causal Relationship between Aging and Anorexia Nervosa: A White-Matter-Microstructure-Mediated Mendelian Randomization Analysis
Source: Biomedicines. 2024 Aug 16;12(8):1874. doi: 10.3390/biomedicines12081874 (PMC11351342; doi:10.3390/biomedicines12081874)
Supplement: Supplementary file 1 [file biomedicines-12-01874-s001.zip › Supplementary Figure/Figure S4/Singe SNP plot of MR analysis for TL to ubm-b-1904.pdf]

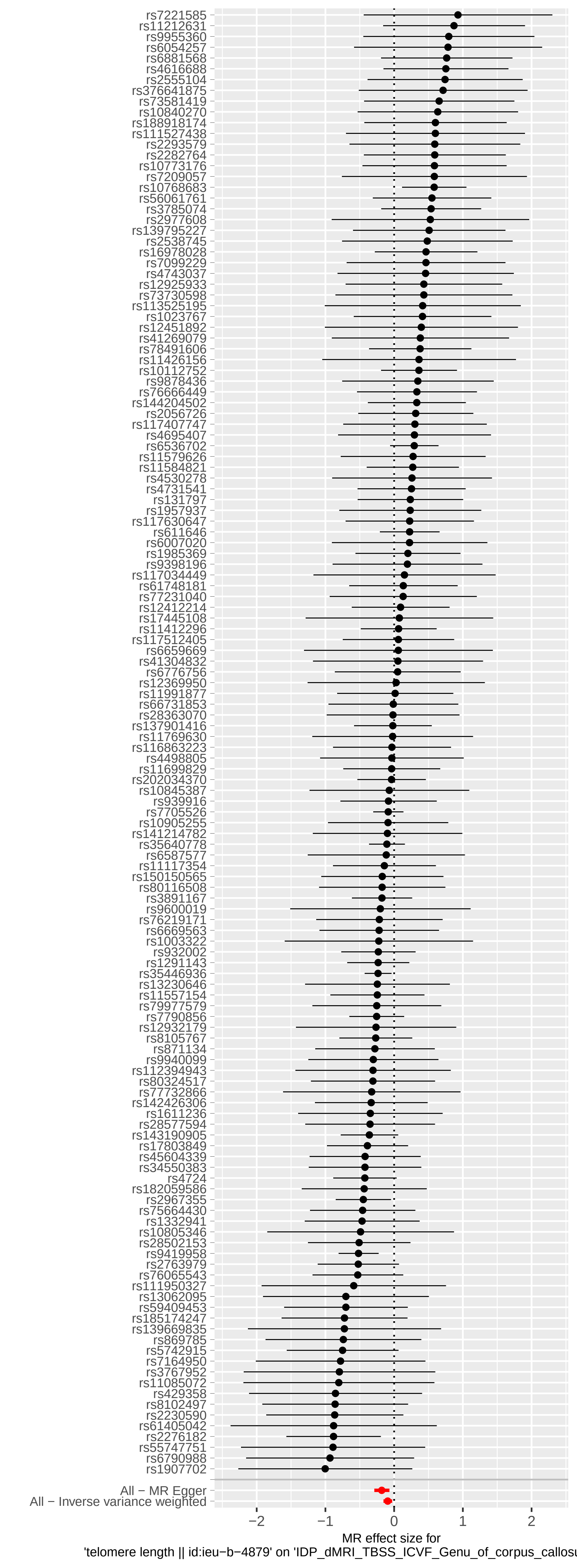
rs7221585
rs11212631
rs9955360
rs6054257
rs6881568
rs4616688
rs2555104
rs376641875
rs73581419
rs10840270
rs188918174
rs111527438
rs2293579
rs2282764
rs10773176
rs7209057
rs10768683
rs56061761
rs3785074
rs2977608
rs139795227
rs2538745
rs16978028
rs7099229
rs4743037
rs12925933
rs73730598
rs113525195
rs1023767
rs12451892
rs41269079
rs78491606
rs11426156
rs10112752
rs9878436
rs76666449
rs144204502
rs2056726
rs117407747
rs4695407
rs6536702
rs11579626
rs11584821
rs4530278
rs4731541
rs131797
rs1957937
rs117630647
rs611646
rs6007020
rs1985369
rs9398196
rs117034449
rs61748181
rs77231040
rs12412214
rs17445108
rs11412296
rs117512405
rs6659669
rs41304832
rs6776756
rs12369950
rs11991877
rs66731853
rs28363070
rs137901416
rs11769630
rs116863223
rs4498805
rs11699829
rs202034370
rs10845387
rs939916
rs7705526
rs10905255
rs141214782
rs35640778
rs6587577
rs11117354
rs150150565
rs80116508
rs3891167
rs9600019
rs76219171
rs6669563
rs1003322
rs932002
rs1291143
rs35446936
rs13230646
rs11557154
rs79977579
rs7790856
rs12932179
rs8105767
rs871134
rs9940099
rs112394943
rs80324517
rs77732866
rs142426306
rs1611236
rs28577594
rs143190905
rs17803849
rs45604339
rs34550383
rs4724
rs182059586
rs2967355
rs75664430
rs1332941
rs10805346
rs28502153
rs9419958
rs2763979
rs76065543
rs111950327
rs13062095
rs59409453
rs185174247
rs139669835
rs869785
rs5742915
rs7164950
rs3767952
rs11085072
rs429358
rs8102497
rs2230590
rs61405042
rs2276182
rs55747751
rs6790988
rs1907702
All − MR Egger
All − Inverse variance weighted
−2
−1
0
1
2
MR effect size for
'telomere length || id:ieu−b−4879' on 'IDP_dMRI_TBSS_ICVF_Genu_of_corpus_callosu
